# Effects of *AMPD1* gene C34T polymorphism on cardiac index, blood pressure and prognosis in patients with cardiovascular diseases: a meta-analysis

**DOI:** 10.1186/s12872-017-0608-0

**Published:** 2017-07-03

**Authors:** Ai-Fang Feng, Zhong-Hui Liu, Shu-Long Zhou, Shi-Yuan Zhao, Yan-Xin Zhu, Huai-Xin Wang

**Affiliations:** Department of Emergency, Weifang Yidu Central Hospital, No. 4138, Linglongshan Southern Road, Weifang, 262500 People’s Republic of China

**Keywords:** *AMPD1*, Cardiovascular diseases, C34T, Polymorphism, Cardiac indexes, Blood pressure, Prognosis

## Abstract

**Background:**

The meta-analysis was aimed to evaluate the effects of *AMPD1* gene C34T polymorphism on cardiac function indexes, blood pressure and prognosis in patients with cardiovascular diseases (CVD).

**Methods:**

Eligible studies were retrieved through a comprehensive search of electronic databases and manual search. Then the high-quality studies met the rigorous inclusion and exclusion criteria, as well as related to the subject was selected for the study. Comprehensive data analyses were conducted using STATA software 12.0.

**Results:**

The study results revealed that CVD patients with CT + TT genotype of *AMPD1* C34T polymorphism presented elevated left ventricular ejection fraction (LVEF) (%) and reduced left ventricular end diastolic dimension (LVEDD) (mm) as compared with CC genotype, moreover, the subgroup analysis found that the LVEF (%) was markedly higher in heart failure (HF) patients carrying CT + TT genotype than CC genotype. Besides, the systolic blood pressure (SBP) (mmHg) in CVD patients with CT + TT genotype was obviously decreased in contrast with the CC genotype. Patients suffered from HF with different genotypes (CT + TT and CC) of *AMPD1* C34T polymorphism exhibited no significant differences in total survival rate and cardiac survival rate.

**Conclusions:**

Our current meta-analysis indicated that the T allele of *AMPD1* gene C34T polymorphism may be correlated with LVEF, LVEDD and SBP, which plays a protective role in the cardiac functions and blood pressure in CVD patients, but had no effects on total survival rate and cardiac survival rate for HF.

## Background

Cardiovascular disease (CVD), a series of diseases involving the heart or blood vessels of people, has become one of the predominant causes of morbidity and mortality worldwide threatening human health according to W.H.O statistics [1–3]. At present, CVD remains the first reason of great number of deaths and disabilities in China, and nearly 3 million individuals died each year from CVD, which approximately accounts for 42% of all deaths in 2010 [4]. In general, CVD, including coronary artery diseases (CAD), heart failure (HF), myocardial infarction and so on, are multifactorial disorders frequently sharing or coexisting risk elements like hypertension, dyslipidemia, overweight, and physical inactivity, which could partially illustrate the etiology of CVD [5, 6]. Apart from the above, mounting evidence has indicated that genetic polymorphisms in some special genes also have important impact on the pathogenesis of CVD, which provided solid foundations to elucidate the essence of clinical phenotype diversity and individual clinical treatment [7, 8].

The *AMPD1* gene encodes the adenosine monophosphate deaminase (AMPD), a crucial enzyme in purine nucleotide and energy metabolism especially in skeletal muscle, as well as in cardiac muscle, with the function of catalyzing adenosine monophosphate to inosine monophosphate [9, 10]. To our knowledge, The *AMPD1* gene is prevalently localized in chromosome 1 in the region p13 ~ 21 with 20 kb spanning in length, and consisting of 16 exons and 15 introns [11]. Under most conditions, there is a common polymorphism of the nonsense mutation with a C-to-T transition at position 34 of *AMPD1*, contributing to the appearance of a premature stop codon, which leads to some related metabolic muscle diseases due to the AMPD activity deficiency [12]. While several recent studies also suggested that *AMPD1* may play essential roles in the initiation and development of CVD, for instance, it has been demonstrated that *AMPD1* allele mutation play a protective role during congestive heart failure (CHF) development, which contributed to prolong possibility of survival without cardiac transplantation [13], meanwhile, it can alleviate the aortic stiffness and inflammation for patients with CAD [10]. Therefore, it was reasonably speculated that *AMPD1* as a possible candidate gene is related to CVD. But the effect of *AMPD1* C34T polymorphism in CVD remains controversial, like Collins RP and his team, who have yielded conflicting results stating that the mutant T allele of *AMPD1*was deleterious in both HF and post-MI patients who had myocardial infarction histories [14]. Besides, de Groote P et al. did not find any impacts of *AMPD1* genetic mutation on the prognosis and survival in CHF patients [15]. Therefore, there appears a need for a meta-analysis to investigate the relationship between the *AMPD1* gene C34T variant and CVD risk regarding the aspects of cardiac indexes, blood pressure and prognosis, and thereby deriving a more plausible estimation for CVD.

## Methods

### Literature research

Published literatures about *AMPD1* gene C34T polymorphism and CVD were retrieved by using following ten English and Chinese databases (including PubMed, Springerlink, Wiley, Web of Science, EBSCO, Ovid, SicenceDirect, Wanfang, China National Knowledge Infrastructure and VIP) from inception through March 2017 with the following search terms: (“*AMPD1*” OR “Adenosine monophosphate deaminase 1”) and (“Cardiovascular disease” OR “CVD” OR “CAD” OR “heart failure” OR “myocardial infarction” OR “heart disease”). In addition, we also carried out a manual search to find potential relevant studies.

### Inclusion and exclusion criteria

The inclusion criteria were as follows: (1) associations of *AMPD1* gene C34T polymorphism with blood pressure, cardiac function and prognosis in CVD patients; (2) studies in which could obtain the parameters (blood pressure, cardiac indexes and prognosis) of CVD patients carrying CT + TT and CC genotypes of *AMPD1* gene C34T polymorphism and calculate WMD (95%CI) and HR (95%CI); (3) unlimited sample size; (4) reliable detection of polymorphic loci; (5) with the same or overlapping data published by the same authors, the most recent or complete articles with the largest sample size; (6) only English or Chinese publications. Literatures that did not conform to the above inclusion criteria or involve one of the following criteria must be eliminated: (1) the literature with only overview and summary (without data) or meeting abstracts; (2) articles with insufficient data; (3) unpublished documents; (4) duplicated publications.

### Data extraction

Two researchers independently searched and reviewed to extract available literature data based on the selection criteria. Disagreements were handled by discussions. The following major information was collected: (1) first author’s name; (2) publication year; (3) age, gender ethnicity and country of subjects; (4) type of diseases; (5) genotyping technology; (6) sample size of the CVD patients carried with different genotypes of *AMPD1* gene C34T polymorphism; (7) LVEF (%), LVEDD (mm), SBP (mmHg), diastolic blood pressure (DBP) (mmHg), total survival rate and the cardiovascular incidence in patients with different genotypes of *AMPD1* gene C34T polymorphism.

### Statistical analysis

Meta-analysis was performed by STATA 12.0 (StataCorp, College Station, TX, USA) software. Kaplan-Meier survival curves were used for HR and 95%CI calculation with the aid of software Engauge Digitizer Version 4.1 (http://sourceforge.net/) and the method [16]. Parameters, such as LVEF (%), LVEDD (mm), SBP (mmHg), DBP (mmHg), total survival rate and cardiovascular incidence in CVD patients with CT + TT and CC genotypes of *AMPD1* gene C34T polymorphism were evaluated using weighted mean difference (WMD) or hazard ratio (HR) and 95% confidence interval (95%CI), and Z test was used to compare the significance of the combined effects [17]. The Cochran’s Q-statistic (*P*_*h*_ < 0.05 was considered significant) and *I* ^*2*^ tests (*I* ^*2*^ > 50%) were used to measure heterogeneity between the studies. The random-effects model was used for significant heterogeneity, while the fixed-effects model was conducted when no significant heterogeneity was existed [18, 19]. Heterogeneity sources were analyzed using subgroup (with or without HF) analysis. The sensitivity analysis was aimed to assess the impact of each single study on the overall estimate. Possible publication bias was tested by Egger’s linear regression analysis [20]. *P* < 0.05 was statistically significant difference.

## Results

### Baseline characteristics of included studies

**Fig. 1:**
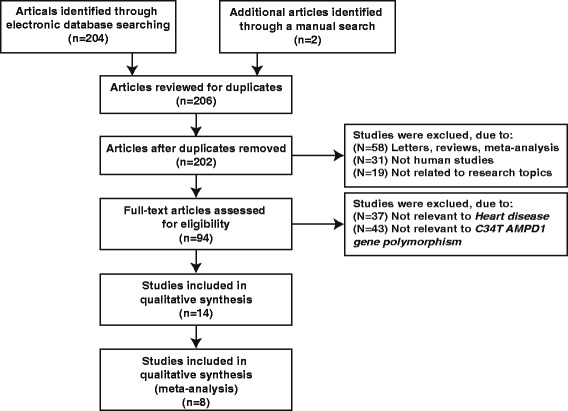
Flow diagram of the literature selection

A total of 206 studies related to *AMPD1* gene polymorphism and CVD (165 English studies and 41 Chinese articles) were retrieved in the initial literature search, of which 108 unrelated articles falling short of titles or abstracts were removed, and the remaining 94 articles were used for further screening. Then, 80 studies of these 94 papers were excluded after more detailed reviewing the full texts, 6 articles with insufficient data and 2 duplicates were removed. Finally, only 8 articles were considered eligible to be incorporated into the meta-analysis [10, 13–15, 21–24]. The flow diagram of the literature selection process is shown in Fig. 1. The included researches which involved 2805 CVD cases all published between 1999 and 2014, including 192 consecutive stable CAD cases, 97 CAD with HF cases, 104 CAD without HF cases, 908 CHF cases, 1395 HF cases and 109 CHD cases. Baseline characteristics of the included studies are shown in Table 1.

**Table 1:** Characteristics of studies included in the meta-analysis

Author	Year	Country	Ethnicity	Disease	Etiology of HF	Genotyping methods	Total number	Number		Gender (M/F)		Age (Year)	
								CC	CT + TT	CC	CT + TT	CC	CT + TT
Tousoulis D [10]	2014	Greece	Caucasian	Consecutive stable CAD		PCR-RFLP	192	149	43	90/10	93/7	61 ± 11	61 ± 11
Safranow K_a [21]	2009	Poland	Caucasian	Stable CAD with HF	Ischemic (67%); Non-ischemic (22%); Other factors (11%)	PCR-RFLP	97	68	29	55/19	27/3	59.92 ± 8.26	56.4 ± 12.0
Safranow K_b [21]	2009	Poland	Caucasian	Stable CAD without HF		PCR-RFLP	104	74	30	55/13	20/9	58.3 ± 8.6	57.9 ± 8.6
de Groote P [15]	2006	France	Caucasian	Stable CHF	Ischemic (42.57%); Non-ischemic (57.43%)	PCR-RFLP	686	517	169	415/102	136/33	55 ± 13	57 ± 13
Collins RP [14]	2006	New Zealand	Caucasian	HF	Irrespective of etiology	PCR-RFLP	357	279	78	167/167	51/48	74.3 ± 0.62	75.4 ± 0.95
Agewall S [22]	2006	Norway	Caucasian	CHD		TaqMan	109	82	27	64/18	18/9	61 ± 11	62 ± 11
Kolek MJ [23]	2005	USA	Mix	HF	Ischemic (58%); Non-ischemic (42%)	PCR-RFLP	1038	843	195	666/177	158/37	60.2 ± 12.2	61.0 ± 12.0
Gastmann A [24]	2004	Germany	Caucasian	CHF	Idiopathic dilated cardiomyopathy (58.89%); Coronary artery disease (22.22%); Other factors (18.89%).	Direct sequencing	90	68	22	51/17	16/6	57.2 ± 13.2	54.0 ± 14.3
Loh E [13]	1999	USA	Caucasian	CHF	Coronary artery disease (52.27%); Idiopathic cardiomyopathy (36.36%); Other factors (11.36%).	ASO-PCR	132	111	21	97/14	16/5	52.1 6 ± 11.6	56.86 ± 7.1

*CAD* Coronary artery disease, *HF* Heart failure, *CHD* Coronary heart disease, *SCHF* Stable congestive heart failure, *CHF* Congestive heart failure, *PCR-RFLP* polymerase chain reaction-restriction fragment Length polymorphism, *ASO-PCR* Allele-specific Oligonucleotide Polymerase Chain Reaction, *Safranow K_a* coronary artery disease in patients with heart failure, *Safranow K_b* coronary artery disease in patients without heart failure

### Correlation of *AMPD1* gene C34T polymorphism with cardiac function indexes (LVEF and LVEDD)

**Fig. 2:**
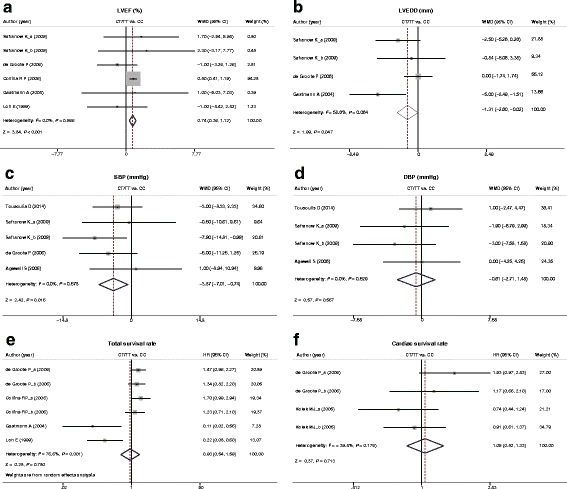
Forest plot for differences in LVEF (%), LVEDD (mm), SBP (mmHg), DBP (mmHg), total survival rate and cardiac survival rate between CVD patients with different genotypes (CT + TT and CC) of *AMPD1* gene C34T polymorphism

**Fig. 3:**
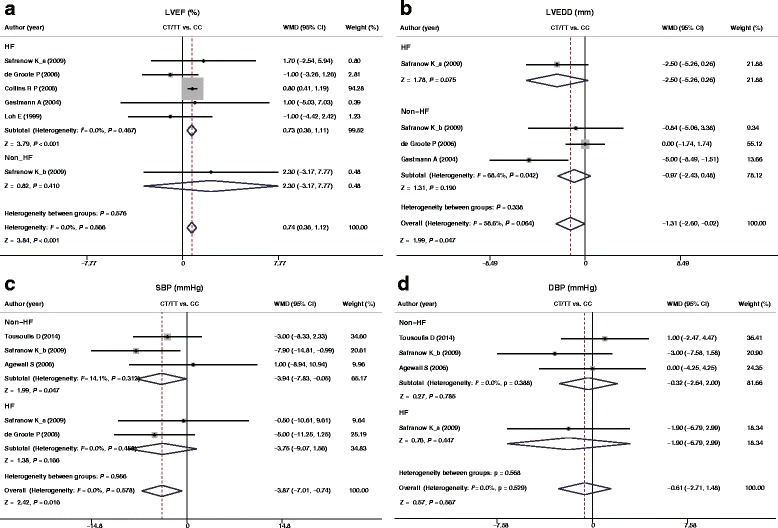
Subgroup analyses for heterogeneity sources in the associations of LVEF (%), LVEDD (mm), SBP (mmHg), and DBP (mmHg) and CVD patients

In this study, no significant heterogeneity was discovered, and accordingly, the fixed effect model was adopted (LVEF: *I* ^2^ = 0.00%, *P*_*h*_ = 0.566; LVEDD: *I* ^2^ = 58.6%, *P*_*h*_ = 0.064). The meta-analysis showed that, as compared with CC genotype, LVEF (%) was significantly increased in CT + TT genotype (WMD = 0.74, 95%CI: 0.36 ~ 1.12, *P* < 0.001, Fig. 2a), while LVEDD (mm) was markedly reduced (WMD = −1.31, 95%CI: -2.60 ~ −0.02, *P* = 0.047, Fig. 2b). The results of subgroup analysis revealed that HF patients with CT + TT genotype had higher LVEF than CC genotype (WMD = 0.73, 95%CI: 0.36 ~ 1.11, *P* < 0.001, Fig. 3a), and, interestingly, LVEDD was turned out to be irrelevant to different genotypes (CT + TT and CC) in CVD patients with or without HF (all *P* > 0.05, Fig. 3b).

### Correlation of *AMPD1* gene C34T polymorphism with blood pressure (SBP and DBP)

No heterogeneity was found among the studies (SBP: *I* ^*2*^ = 0%, *P*_*h*_ = 0.578; DBP: *I* ^*2*^ = 0%, *P*_*h*_ = 0.529), and accordingly, the fixed effect model was used. SBP (mmHg) was significantly lower in CVD patients with the CT + TT genotype compared with CC genotype by meta-analysis (WMD = −3.87, 95%CI: -7.01 ~ −0.74, *P* = 0.016, Fig. 2c), however, there were no such differences in DBP (mmHg) (WMD = −0.61, 95%CI: -2.71 ~ −1.48, *P* = 0.567, Fig. 2d). Subgroup analysis demonstrated that different genotypes (CT + TT and CC) of *AMPD1* gene C34T polymorphism in CVD patients with or without HF had no impact on SBP and DBP (both *P* > 0.05, Fig. 3c, d).

### Effects of *AMPD1* gene C34T polymorphism on total survival rate and cardiac survival rate

Effects of *AMPD1* gene C34T polymorphism on total survival rate were studied in 4 literatures, and it was considerably heterogeneous among the 4 studies (*I* ^*2*^ = 76.6%, *P*_h_ = 0.001), thus the random effects model was selected. Two studies were about the effects of *AMPD1* gene C34T polymorphism on cardiac survival rate, and it was proved to be with heterogeneity (*I* ^*2*^ = 39.4%, *P*_h_ = 0.176), and the fixed effect model was preferred. Among 4 studies, two suggested that *AMPD1* gene C34T polymorphism of (CT + TT) enhanced the total survival rate of patients, while the other two found that it had no effects on the total survival rate and cardiac survival rate. Meta-analysis results showed that there were no distinctly differences in total survival rate and cardiac survival rate in CAD patients with different *AMPD1* gene C34T polymorphism genotypes (Total survival rate: HR = 0.93, 95%CI: 0.54 ~ 1.59, *P* = 0.780; Cardiac survival rate: HR = 1.05, 95%CI: 0.82 ~ 1.33, *P* = 0.713, Fig. 2e, f).

### Sensitivity analysis and publication bias

**Fig. 4:**
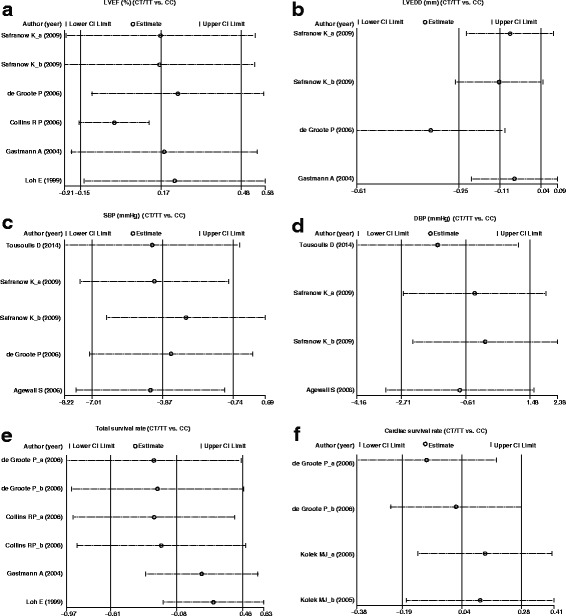
Sensitivity analysis of the relationship between *AMPD1* gene C34T polymorphism in patients with different genotypes (CT + TT and CC) and LVEF (%), LVEDD (mm), SBP (mmHg), DBP (mmHg), total survival rate and cardiac survival rate

**Fig. 5:**
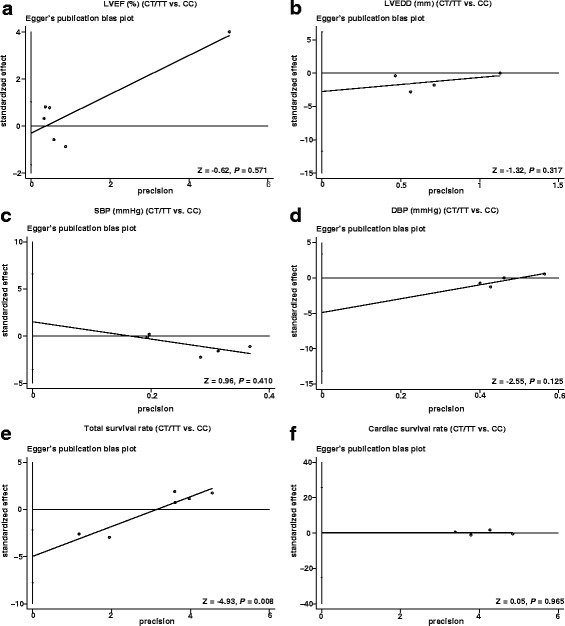
Egger’s test for assessing the publication bias of included studies by comparing LVEF (%), LVEDD (mm), SBP (mmHg), DBP (mmHg), total survival rate and cardiac survival rate between patients with different genotypes (CT + TT and CC) of *AMPD1* gene C34T polymorphism

Sensitivity analysis verified that all included studies presented no evident impacts on pooled WMD (Fig. 4a-d), as well as the pooled HR (Fig. 5e, f). Egger linear regression analysis indicated that there were no publication bias in the correlation of different genotypes (CT + TT and CC) of the *AMPD1* gene C34T polymorphism with cardiac indexes (LVEF and LVEDD), blood pressure (SBP and DBP) and cardiac survival rate (all *P* < 0.05, Fig. 5a-e), while the total survival rate had a publish bias (*P* = 0.008, Fig. 5f).

## Discussion

The present meta-analysis has combined and reanalyzed 8 publications involving 2805 cases of patients with CVD, to assess the effect of *AMPD1* gene C34T polymorphism on LVEF (%), LVEDD (mm), SBP (mmHg), DBP (mmHg), total and cardiac survival rate of CVD.

Our study showed that LVEF (%) in CVD patients with T allele of *AMPD1* gene C34T polymorphism was significantly increased in comparison to those patients with CC genotype. On the contrary, the CVD patients with CT + TT genotype had a greater reduction of LVEDD (mm) than those with CC genotype. In addition, our subgroup analysis according to the patients with or without HF verified that the LVEF was markedly higher in HF patients who carried with CT + TT genotype than CC genotype. As is known to all, abnormalities of left ventricle (LV) function or structure has been a common event among most CVD patients, demonstrating a negative influence on cardiovascular prognosis [25, 26]. While LVEF is well-recognized as an important determinant of a person’s cardiac function to be clinically applied for the evaluation of the severity of heart systolic function, and in particular, it is typically low when the patients with the systolic form of congestive heart failure [27]. As for LVEDD, it has widely accepted as a sensitive indication for measurement of the left ventricular remodeling for patients with CVD [28]. More importantly, the myocardial remodeling, which is marked by changes in LVEF and LV volumes, could be functioned as a practical prognosis indicator to guide clinicians’ diagnose [29]. Probably, there is an explanation for this finding that this polymorphic variant of *AMPD1* C34T could weaken the AMPD activity, and promote the elevated circulating levels of adenosine, a crucial protective agent, which was in part the consequence of the attenuation of myocardial fibrosis and ventricular remodeling, and thus contributing to less severe CVD [13, 30]. Consistently, both Safranow K et al. [21] and Gastmann A et al. [24] pointed out the tendency that LVEDD values in patients with CAD or CHF who carried the mutant T allele of *AMPD1* was smaller, further indicating that the *AMPD1* C34T polymorphism might affect different types of ventricular remodeling among CVD. Interestingly, no relevance of *AMPD1* gene C34T polymorphism to LVEF (%) had been found in our 5 included researches [13–15, 21, 24], which might be partially limited by the small sample size. To our knowledge, high blood pressure (BP), or together with other risk elements could maximize the cardiovascular risk to a certain extent [5]. In the present study, we also revealed that being carrier of the T allele in *AMPD1* C34T polymorphism is remarkably correlated with lower levels of SBP, indicating that T allele carriers of this SNP may benefit for CVD individuals, as it have a decreased risk of developing the CVD pathologic state through a lower value of SBP. As shown by Safranow K*,* T allele carriers tended to have lower SBP than wild type in patients with CAD and HF, especially in ischemic HF patients, what might account for the lower fasting serum glucose or the higher creatinine clearance due to the T allele in patients with HF [21]. Moreover, fasting serum glucose has been conformed to be positively correlated with SBP in a previous research [31]. Therefore, the relatively increased LVEF and reduced LVEDD (or LV volumes) in CVD patients indirectly indicated that the T mutant allele of *AMPD1* variant performed a protective effect on cardiac function recovery.

In addition, the present meta-analysis further explored the possibility of a relative survival and cardiovascular incidence benefit for the *AMPD1* genetic mutation in HF patients, but failed to find any significant differences between different genotypes (CT + TT and CC) of this polymorphism and total survival rate and cardiac survival rate. It may therefore be possible that adenosine concentration in plasma (or muscle) of different genotypes showed no differences although T allele could induce the synthesis of myocardial adenosine [32, 33]. Moreover, the adenosine concentration in patients with T allele was only markedly increased after exercise as suggested previously, hence, the protective role of T allele might exert protective functions through increasing the local secretion of adenosine due to the short half-life of adenosine [34, 35]. Our result was broadly consistent with Kolek MJ group, who reported the *AMPD1* genotype had no statistically influences on the survival of HF [23]. Similarly, no influence of the functional AMPD1 C34T polymorphism has been demonstrated by De Groote P et al. on the major clinical parameters and cardiac survival in consecutive white patients with stable CHF linked to left ventricular systolic dysfunction [15].

There are several limitations of the current meta-analysis need to be pointed out. First, as we know, heart failure can be caused by atherosclerotic and non-atherosclerotic factors, but we couldn’t conduct a subgroup analysis due to the fact that the included studies didn’t analyze the effects of AMPD1 gene C34T polymorphism on cardiac index, blood pressure and prognosis in patients with HF based on the etiology.. Second, heterogeneity in our study may influence the reliability of our results, although subgroup analysis was conducted to detect the heterogeneity source, as well as sensitivity analysis was introduced to appraise the stability of the results. Third, the data extracted from each record were based on unadjusted estimates, which may lead to misleading results. Fourth, we did not obtain all desired information from all materials because of the small number of studies. Fifth, the relatively small sample size of our study might have negative impact on the power of our meta-analysis. Lastly, the limited English and Chinese studies might also lead to language bias despite no evidence of publication bias from our statistical tests.

## Conclusion

In conclusion, our meta-analysis suggested that T allele of *AMPD1* gene C34T polymorphism was correlated with LVEF (%), LVEDD (mm) and SBP (mmHg) of CVD, which plays a protective role in the recovery of ventricular remodeling and cardiac function, as well as blood pressure, but has no influences on the total survival rate and cardiac survival rate of HF patients. Further well-designed researches with large sample size and complete clinical data are needed to test and verify our findings.

## Abbreviations

AMPD1: adenosine monophosphate deaminase 1
CAD: coronary artery diseases
CHF: congestive heart failure
CVD: cardiovascular diseases
DBP: diastolic blood pressure
HF: heart failure
LVEDD: left ventricular end diastolic dimension
LVEF: left ventricular ejection fraction
SBP: systolic blood pressure
